# Review of the Predictive Value of Biomarkers in Sepsis Mortality

**DOI:** 10.1155/2024/2715606

**Published:** 2024-06-05

**Authors:** Nai Zhang, Yujuan Liu, Chuang Yang, Xinai Li

**Affiliations:** ^1^Department of Emergency, Jiangxi Province Hospital of Integrated Chinese and Western Medicine, Nanchang 330003, China; ^2^Department of Respiratory Medicine, Jiangxi Province Hospital of Integrated Chinese and Western Medicine, Nanchang 330003, China

## Abstract

Sepsis is a leading cause of mortality among severely ill individuals, primarily due to its potential to induce fatal organ dysfunction. For clinicians, it is vital to have appropriate indicators, including the physiological status and personal experiences of patients with sepsis, to monitor the condition and assess prognosis. This approach aids in preventing the worsening of the illness and reduces mortality. Recent guidelines for sepsis focus on improving patient outcomes through early detection and timely treatment. Nonetheless, identifying severe cases and predicting their prognoses remain challenging. In recent years, there has been considerable interest in utilising the C-reactive protein (CRP)/albumin ratio (CAR) to evaluate the condition and forecast the prognosis of patients with sepsis. This research concentrates on the significance of CAR in the pathological process of sepsis, its association with prognosis, and the latest developments in employing procalcitonin, lactic acid, CRP, and other potential biomarkers. The CAR, with its predictive value for sepsis prognosis and mortality, is increasingly used as a clinical biochemical marker in diagnosing and monitoring patients with sepsis.

## 1. Introduction

In 2001, the Society of Critical Care Medicine (SCCM), the American College of Chest Physicians, and the European Society of Intensive Care Medicine (ESICM) convened a joint meeting in Washington to revise the original sepsis criteria, termed Sepsis 1.0. Although infection and systemic inflammatory response syndrome (SIRS) remained foundational elements, Sepsis 2.0 introduced notable improvements in diagnostic standards. Specifically, 21 new diagnostic indicators were added, encompassing infection or suspected infection, inflammatory response, organ dysfunction, and hemodynamic or tissue perfusion markers [1]. In January 2014, a panel of 19 experts from the SCCM and ESICM specialising in infection and epidemiology was assembled to refine the definition and diagnostic criteria of sepsis further. This effort culminated in the release of Sepsis 3.0 in February 2016. The revised definition characterises sepsis as a life-threatening organ dysfunction resulting from the host's dysregulated response to infection [2].

Sepsis has consistently been a leading cause of death in critically ill patients worldwide. Defined as an organ dysfunction that poses a life-threatening risk, sepsis arises from infection and a dysregulated host response [3]. According to the 2017 Global Burden of Diseases, Injuries, and Risk Factors Study, sepsis was responsible for an estimated 48.9 million incident cases and 11 million deaths globally in 2017 [4]. Despite considerable advances in treatment, sepsis continues to exhibit high mortality rates and places substantial burdens on healthcare systems. Prompt identification and initiation of therapy are essential for mitigating adverse outcomes.

The current gold standard for diagnosing sepsis involves culturing pathogens from patients' body fluids, such as blood and cerebrospinal fluid, and administering antibiotics. However, this method has limitations, including lengthy culture times and high rates of false negatives. The complexity of sepsis and the variability of infections in patients present additional challenges. Sepsis 3.0 emphasises the host's uncontrolled response to infection, which can precipitate organ failure. It advocates for the Sequential Organ Failure Assessment (SOFA) score as a reliable metric for assessing organ dysfunction and determining the severity of the condition. Nonetheless, SOFA's extensive criteria limit its therapeutic applicability. Previously, the Acute Physiology and Chronic Health Evaluation (APACHE) II score and other less precise severity assessment tools were employed for evaluating the severity of sepsis. These tools offer a broad range of indicators, but their ability to assess organ function is limited. In practical application, they are cumbersome and imprecise, potentially delaying the treatment of patients with sepsis and impacting disease progression [5].

Sepsis biomarkers could offer data inaccessible through other tests, assisting clinical judgement and potentially improving patient care. However, none of the detected biomarkers have shown “sufficient specificity or sensitivity to be routinely utilised in clinical practice” [6]. Early identification of patients at risk of sepsis allows for appropriate treatment to commence upon admission to the intensive care unit (ICU), potentially leading to improved outcomes [7]. While each biomarker has its drawbacks, most are useful for diagnosing specific illnesses. Recent years have seen a growing focus on researching biomarkers for sepsis detection. The prediction of sepsis has been enhanced by biological indicators, such as procalcitonin (PCT), lactate, C-reactive protein (CRP), cytokines, D-dimer, proadrenomedullin (proADM), and cardiac biomarkers [8]. C-reactive protein and PCT are crucial in the early diagnosis, precise individualised treatment, and prognosis of patients with sepsis. In diagnosing newborns with late-onset specific infections, CRP showed a slow increase at the onset of infection. After controlling the infection, the index returned to normal, serving as a marker for discontinuing antimicrobial therapy. Furthermore, a reduction in CRP levels by 25% or more from the previous day's level is a strong indicator of sepsis resolution. However, CRP measurements do not differentiate between systemic inflammatory responses and sepsis. Research also revealed a correlation between increased PCT levels and sepsis, albeit only 79% [9]. Despite extensive research, no biomarkers have yet demonstrated sufficient sensitivity and specificity for routine clinical use. Sepsis is complex; thus, a single biomarker cannot fulfil all research and management needs. To overcome the limitations of relying on a single biomarker, researchers have proposed using a combination of biomarkers to represent different aspects of the host's response. Recent studies have focused on integrating certain biomarkers with criticality scoring systems, aiming to enhance sensitivity and specificity [10]. Recent research highlights the predictive significance of the CRP/albumin ratio (CAR), an innovative inflammation-based prognostic score that combines a positive acute reactant protein with a negative acute reactant protein [6, 11].

This study reviews the characteristics and properties of common biological markers in sepsis mortality, primarily focusing on the predictive value of CAR. It presents the development and implementation of CAR as a mortality predictor in sepsis.

### 1.1. The Current Status of Sepsis Mortality Prediction

#### 1.1.1. Epidemiology

Sepsis is categorised into adult and neonatal sepsis. Based on population-level research conducted over the past two decades, sepsis fatality rates worldwide are estimated to be between 11% and 19% [12]. Each year, more than 30 million individuals globally are affected by adult sepsis. This condition is the leading cause of mortality in noncardiac ICUs and ranks among the primary causes of death in critically unwell patients [13, 14]. Most data on the prevalence of sepsis are derived from high-income countries, where adult sepsis accounts for approximately 2.8 million deaths annually [15].

A meta-analysis covering 27 studies from seven high-income countries reported an incidence rate of severe sepsis in adults of 270 per 100,000 person-years, accompanied by a case-fatality ratio of 26% [16]. According to the 2017 Global Burden of Diseases, Injuries, and Risk Factors Study, sepsis was responsible for 11 million deaths and 48.9 million incident cases worldwide in that year. Areas with the lowest sociodemographic index exhibited the highest age-standardised incidence and mortality rates of sepsis [2].

Sepsis also represents the most prevalent cause of neonatal morbidity and mortality, incurring an annual cost of $1.97 billion and accounting for 7% of infant fatalities in the United States. The United Nations has reported that severe sepsis is responsible for 17% of child mortality in neonatal ICUs. Globally, sepsis contributes to 20% of the 3 million newborn deaths each year. Notably, sub-Saharan Africa experiences the highest neonatal sepsis mortality rates, in contrast to the situation in high-income countries. Between 1996 and 2015, China experienced a considerable decline in its newborn sepsis mortality rate, from 0.4 to 0.1 per 1,000 live births, compared with the 2015 global estimate of 2.8 per 1,000 live births [17].

## 2. Biological Markers Predicting Sepsis Mortality

### 2.1. Routine Laboratory Tests

#### 2.1.1. Procalcitonin

Procalcitonin is a 116-amino-acid glycoprotein and the mRNA product of the CALC-1 gene, located on chromosome 11 in healthy individuals, and is produced in thyroid C cells. However, during inflammation, two main pathways are activated: a direct pathway, initiated by lipopolysaccharides or other harmful bacterial metabolites, and an indirect pathway, induced by numerous inflammatory mediators, such as interleukin (IL)-6 and tumour necrosis factor-alpha [18]. In patients with sepsis, the stimulation of inflammatory cytokines leads to tissues other than the thyroid—such as the liver, kidney, lung, intestine, fat, and muscle—synthesising and secreting PCT. This results in a notable increase in serum PCT levels, which positively correlates with the severity of the illness [19].

Several studies have demonstrated the utility of PCT as a promising tool for the early detection of sepsis, monitoring antimicrobial treatment regimens, and ultimately decreasing sepsis mortality. Notably, it is the only inflammatory factor included in the sepsis diagnosis and treatment guidelines by the German Society for Emergency and Critical Care [20]. Evdoxia's research [21] investigated the impact of PCT advice in reducing the emergence of long-term infection-related negative consequences in sepsis. In this multicentre study, 266 patients were randomised (1 : 1) to receive either standard-of-care (SOC) or PCT-guided cessation of antibiotics, depending on whether they had acute pyelonephritis, lower respiratory tract infections, or main bloodstream infections. The results indicated that the PCT-guided arm had a lower 28-day mortality rate compared with the SOC arm (15.2% [19/125 patients] vs 28.2% [37/131 patients]; hazard ratio = 0.51; 95% confidence interval [CI] = 0.29–0.89; *p*=0.02). The study also highlighted that PCT guidance contributed to lower inpatient costs and reduced death rates and adverse infection-related events at 28 days. According to a meta-analysis [22] of individual patient data from 11 randomised trials involving 4,482 patients with infections treated in ICUs, the mortality rate in the 2,252 PCT-guided patients was significantly lower compared with the 2,230 control group patients (21.1% vs 23.7%; adjusted odds ratio [OR] = 0.89, 95% CI = 0.8–0.99; *p*=0.03).

However, in cases involving trauma, burns, or heart surgery, high concentrations of PCT can be detected, suggesting a limitation in its specificity. Studies questioning the efficacy of PCT in diagnosing and prognosticating sepsis have noted discrepancies between the severity of illness and infection and PCT predictions. Consequently, the widespread use of PCT as a diagnostic test for critically ill patients is not recommended.

#### 2.1.2. C-Reactive Protein

C-reactive protein is a nonspecific inflammatory marker extensively utilised in clinical practice. It is a pentameric protein with a molecular weight of 23 kDa, first identified in 1930 by Tillet and Francis during their investigation into the sera of patients with acute Pneumococcus infection [23]. C-reactive protein is a highly sensitive, real-time indicator of inflammation, capable of increasing up to 1,000-fold at sites of infection or inflammation [24, 25]. Li's [26] findings indicate that CRP levels were considerably higher in nonsurvivors than in survivors, showing a positive correlation with the severity of sepsis as measured by the SOFA score and the partial pressure of oxygen/fraction of inspired oxygen ratio. Moreover, multivariate analysis demonstrated that CRP levels within the first 24 hours of admission independently predicted the 28-day mortality rate in patients with sepsis. A 2019 meta-analysis assessing the clinical utility of both PCT and CRP in diagnosing adult sepsis attributed a moderate diagnostic value to CRP [27]. Identifying shifts in CRP patterns can assist general practitioners in the early detection of sepsis. However, it is important to acknowledge that postsurgical SIRSs and noninfectious conditions such as autoimmune and rheumatic diseases, myocardial infarction, and malignant tumours can also elevate CRP levels. Despite its widespread use in clinical practice, CRP lacks specificity in infection diagnosis, with factors like bacterial and viral infections, acute rejection, surgery, and cardiovascular diseases leading to elevated CRP levels.

#### 2.1.3. Lactate

The L-enantiomer of the lactate anion is understood to be continuously created and utilised in various cells, even under fully aerobic conditions [28]. Lactate, a molecule produced during the Warburg effect, was previously believed to result solely from oxygen deficiencies in contracting skeletal muscle [28]. Hyperlactatemia is known to predict a notable fatality rate in patients with sepsis.

Arterial blood lactate clearance is a sensitive and accurate measure for assessing and predicting sepsis mortality [29]. It is now understood that one of the primary contributors to hyperlactatemia in patients with sepsis is accelerated aerobic glycolysis triggered by adrenergic stress. Other contributing factors include poor clearance, drug side effects, microcirculatory dysfunction, and tissue hypoperfusion [30, 31]. A study by Ryoo et al. [32] involving patients with septic shock who met the Sepsis 3.0 diagnosis criteria included 1,060 patients; of these, 265 died (28-day mortality rate: 25%). The median 6-hour lactate levels in the survivor group were lower than those in the nonsurvivor group, and their lactate clearance rates were higher (2.5 vs 4.6 mmol/L and 35.4% vs 14.8%, respectively; *p* < 0.01). After adjusting for covariates, both lactate and lactate clearance were associated with mortality (OR: 1.27 [95% CI 1.21–1.34] and 0.992 [95% CI 0.989–0.995], respectively), although lactate was found to be more predictive of mortality than lactate clearance (area under the curve = 0.70 vs 0.65; *p* < 0.01). This study suggests that targeting both lactate and lactate clearance can be beneficial for individuals with septic shock, as defined by Sepsis 3.0.

#### 2.1.4. D-Dimer

D-dimer, a soluble fibrin degradation product, serves as a biological index often utilised to measure the intensity of inflammation. Extensive research has been conducted on the application of D-dimer in diagnosing venous thromboembolism (VTE). It is now frequently used for this purpose. D-dimer has also been investigated for determining the optimal duration of anticoagulation in patients with VTE, detecting and monitoring disseminated intravascular coagulation and identifying individuals at high risk of VTE [33].

Sepsis is a risk factor for VTE [34]. The pathophysiology of VTE in sepsis is not fully understood. However, it is thought that several variables, including immobility, activation of thrombotic and inflammatory pathways, disseminated intravascular coagulation, and venous stasis, contribute to the condition. Diagnosing VTE in patients with sepsis can be challenging, potentially leading to underdiagnosis and subsequent consequences, such as hypotension, tachycardia, hypoxia, and lung damage [35]. A prospective study by Kaplan et al. [36] involving 113 consecutively enrolled ICU patients with severe sepsis and septic shock across three hospitals revealed a substantially higher (approximately 3–10 times) incidence of VTE in patients with sepsis compared with those admitted to the ICU for other reasons. However, although the 28-day mortality of sepsis was numerically higher in patients with severe clinical VTE, this observation did not reach statistical significance.

### 2.2. Specific Tests

#### 2.2.1. Cytokines

Cytokines are polypeptides or glycoproteins, typically with a molecular weight below 30 kDa, that signal various cell types for growth, differentiation, and pro- or anti-inflammatory responses [37]. Pro-inflammatory ILs, including IL-1, IL-6, IL-12, and IL-17, are primarily responsible for cell activation, tissue damage, and necrosis [38–41]. Anti-inflammatory ILs, such as IL-1RA, IL-4, and IL-10, aim to mitigate and ultimately reverse the inflammatory process [42–44]. Several studies have linked the levels of IL-6 and IL-10 with the mortality of patients with sepsis, with IL-6 emerging as an independent risk factor for 28-day mortality [45]. Similarly, Chuang et al. [46] found that an increase in blood IL-10 concentration in patients with severe early-stage sepsis directly correlated with a higher mortality risk at 48 hours.

#### 2.2.2. Proadrenomedullin

As a precursor fragment of adrenomedullin, proADM is commonly used as a novel index to predict sepsis in clinical practice. Its level in serum is proportional to the severity of the infection; the higher the proADM expression level, the more severe the disease [47]. Adrenomedullin is similarly elevated in sepsis but rapidly converts into a smaller molecule, proADM [48]. This vasodilatory peptide, known for its antibacterial and anti-inflammatory effects, has proven effective in predicting the severity of sepsis [49, 50]. According to Angeletti et al. [51], proADM was more accurate in predicting death than PCT, and proADM levels were substantially higher in severe sepsis compared with sepsis alone.

#### 2.2.3. Myocardial Biomarkers

Septic cardiomyopathy, often observed in severe sepsis syndromes, is characterised by widespread cardiomyocyte death, reduced ejection fraction, and interstitial oedema [52]. Cardiac dysfunction is a major contributor to sepsis-induced multi-organ failure in critical care settings [53], and diagnostic methods include the identification of biomarkers, such as troponin and natriuretic peptides [54]. The Albumin Italian Outcome Sepsis trial, a multicentre randomised clinical trial, focused on albumin replacement in severe sepsis or septic shock. It was found that concentrations of *N*-terminal pro-B-type natriuretic peptide better predicted ICU or 90-day mortality than high-sensitivity cardiac troponin T [55]. Early variations in high-sensitivity cardiac troponin T or *N*-terminal pro-B-type natriuretic peptide concentrations were independently associated with subsequent mortality in patients with shock. According to Yao's research [56], higher myoglobin levels in patients with sepsis correlated positively with their SOFA score, CRP level, and PCT level. Elevated myoglobin levels might exacerbate oxidative stress-related damage [57]. Additionally, the mortality of patients with sepsis was linked to elevated myoglobin levels, indicating increased oxidative stress.

#### 2.2.4. MicroRNA

MicroRNA, a type of noncoding RNA comprising 20–25 nucleotides, primarily influences the expression of functional genes to regulate the development and progression of sepsis. This regulation includes aspects of the inflammatory response, immune cell differentiation and death, and immunosuppression. Circulating microRNA can serve as a biomarker for diagnosing sepsis and assessing its prognosis [58]. According to a meta-analysis, the levels of microRNA, particularly miR-223, can be used as a diagnostic marker for sepsis [59]. Concurrently, several studies have shown that the expression levels of microRNAs 146a, 150, 233, 486, 182, 340, 324-3P, 16, 210, 15b, 484, and 486-5p considerably change in patients with sepsis following multiple injuries, suggesting their potential as biomarkers for diagnosing and prognosticating posttraumatic sepsis [60].

#### 2.2.5. CD4+CD25+ Regulatory T Cells

In 1995, Sakaguchi et al. discovered regulatory T (Treg) cells in mice. These cells primarily play an inhibitory role in cellular immunity, specifically within the complex immune regulatory network of sepsis. During sepsis, the level of CD4+CD25+ Treg cells in the body increases, thereby exacerbating the immune response. Studies have demonstrated that patients with sepsis possess excessive Treg cells, which can induce lymphocyte apoptosis through cell contact mechanisms and the release of cytokines, such as IL-10 and transforming growth factor-*β*. These cells also downregulate the expression of CD80/CD86 on the surface of dendritic cells, inhibit the function of CD4+ and CD8+ T cells, and affect the polarisation of Th1/Th2, determining the outcomes of different inflammatory responses [61]. Research indicates that individuals with septic shock have notably more CD4+CD25+ Treg cells compared with those in the sepsis, severe sepsis, and sepsis groups combined [62]. As the severity of the condition increases, the production of CD4+CD25+ Treg cells escalates, leading to a decrease in CD4+ and CD4+/CD8+ ratios and enhanced inhibition of immune function in patients with sepsis, ultimately resulting in immunological paralysis. However, current research on CD4+CD25+ Treg in sepsis, especially in clinical observations, is still limited, and numerous issues, such as the precise regulatory mechanism, require further investigation.

### 2.3. C-Reactive Protein/Albumin Ratio

#### 2.3.1. Introduction of the C-Reactive Protein/Albumin Ratio

C-reactive protein is a sensitive index for measuring bodily injury and infection, while albumin is an important indicator of the body's nutritional status. During the acute phase reaction, inflammation-related factors cause a rise in CRP and a decrease in albumin [63]. However, as individual indicators of inflammation, measurements of CRP and albumin alone are not as effective as CAR. This well-established scoring system is used to assess the severity and activity of inflammatory illnesses [64]. This novel biomarker integrates the responses of CRP and albumin to systemic inflammatory responses and dystrophy [6]. A high CAR is associated with more advanced disease stages and worse clinical outcomes, either as independent parameters or as components of other prognostic scores. It was initially reported for identifying patients with serious illnesses in acute medical wards [65–67].

To date, the CAR has been linked with the severity and poor outcomes of various conditions, including sepsis [7], cancer [68–73], cardiovascular diseases [74, 75], abdominal aortic aneurysm [76], aneurysmal subarachnoid haemorrhage [77], AIDS-related pneumocystis pneumonia [78], acute pancreatitis [79], carotid artery stenosis [80], Guillain–Barré syndrome [81], Bell's palsy [82], and primary and secondary myelofibrosis [83]. Notably, the CAR is simpler to calculate and easier to obtain than other rating standards, making it a crucial tool for quickly assessing patients' conditions in emergency environments [84, 85]. Although the detection of serum CRP and albumin alone is less sensitive than the CAR in determining the prognosis of severe cases, the critical value of a high CAR has not been standardised. Furthermore, exploration through future multicentre prospective studies is needed.

#### 2.3.2. Use of C-Reactive Protein/Albumin Ratio in Predicting Sepsis Mortality

Studies have demonstrated CAR's ability to predict death related to sepsis. Otavio's [86] retrospective analysis of prospectively collected data from 334 patients admitted to the ICU due to severe sepsis or septic shock—and discharged alive after a minimum of 72 hours—indicates that the CAR at discharge more accurately predicts mortality at 90 days than does CRP alone at discharge. Furthermore, the model including CAR at discharge showed better calibration for predicting long-term mortality in critically ill patients with sepsis than the model with only CRP levels at discharge. In Ayranci's [87] retrospective observational study, which included 784 participants, the in-hospital mortality rate of geriatric patients with concurrent high CAR (>12.3) values during admission was statistically significantly higher than in those without such high values. This comparison revealed increased CAR values to be statistically significant in the group with mortality compared with the group without mortality (21.39 [95% CI 6.02–55.07] vs 4.82 [95% CI 1.17–17.03]; *p* < 0.001). Raim's [88] trial linked an elevated inflammatory response to higher fatality rates.

Patients with sepsis are in a state of high catabolism and hypermetabolism; they also expend considerable energy in the resting state. The metabolism and decomposition of fat and protein increase, often leading to malnutrition. Concurrently, the immune response to infection can increase basal metabolism and exacerbate malnutrition. Malnutrition may lead to immunosuppression, impairing infection control. This interaction between poor infection control and malnutrition creates a vicious cycle. Therefore, an increase in CAR indicates a severe patient condition and suggests a poor prognosis [89]. According to Tak's study [84], variations in cut-off values across studies reflect different patient characteristics in each study. Hence, it is vital to establish CAR's reference value while considering the characteristics of the study population. Patients with sepsis often exhibit higher levels of inflammatory markers such as CRP or PCT [90], leading to larger CAR values than those observed in previous studies.

#### 2.3.3. Why Can the C-Reactive Protein/Albumin Ratio Be a Predictor of Mortality in Sepsis?

Sepsis, the leading cause of mortality in ICUs, was declared a global health priority by the World Health Organization in 2017 [91]. Annually, it affects 31.5 million individuals, resulting in 6 million deaths and leaving 3 million with disabilities that necessitate hospital readmission postsepsis [92]. Although infection is the primary cause of sepsis, the dysregulated immune response persists long after adequate treatment of the infection [1], despite sepsis being officially defined as life-threatening organ failure caused by a dysregulated host response to infection [10]. Due to substantial patient heterogeneity, early identification of individuals at high risk of mortality is critical. Prompt and appropriate decisions regarding therapeutic approaches are imperative for enhancing survival and minimising in-hospital mortality rates.

Patients with sepsis often experience severe metabolic stress. Even if patients had no history of malnutrition during their initial hospital stay, the inflammatory process, instigated by the underlying disease and/or its sequelae, may exacerbate their nutritional status [93, 94]. During this process, there is a reduction in serum albumin synthesis and an increase in acute phase proteins, such as CRP [95]. The CAR is a parameter of growing interest. Prior research indicates that CAR can predict morbidity, mortality, and other outcomes, including sepsis, in various populations [96].

The CAR is calculated by dividing the CRP by the albumin value. A nonspecific acute phase response protein produced by the liver, and CRP aids in the activation of the C1q complex in the complement system. During inflammation and infection, CRP levels rise within 2 hours and peak at around 48 hours. It is a widely utilised index for evaluating inflammatory responses [97]. Hypoalbuminemia is recognised as a predictive factor in elderly hospitalised patients, and serum albumin level is an indicator of malnutrition [98]. Given their capacity to signify acute inflammatory responses and malnutrition in critically ill patients, including those with sepsis, CRP and serum albumin levels are often employed to predict mortality [99, 100]. However, due to their association with various illnesses, changes in CRP and albumin levels may lack specificity. In contrast, CAR offers a more consistent predictive value and more accurately reflects the severity of nutritional deficiencies and inflammation. The CAR, a straightforward laboratory test, utilises common parameters that are easy to obtain, calculate, and remember.

## 3. Summary

In conclusion, sepsis is a prevalent, life-threatening condition associated with high mortality and considerable healthcare costs [101]. Predicting mortality in sepsis and treating high-risk patients early to provide timely and appropriate therapy is crucial for improving survival rates. The SOFA and APACHE-II scoring systems [102–105] are used as indicators of mortality in sepsis. Although moderate progress has been made in the field of biomarkers, most research has focused on single indicators. However, given the complexity of the sepsis response, the primary focus should shift towards combinations of markers [106]. Biomarkers have the potential to improve future outcomes by aiding in the prediction of sepsis mortality, reducing the time to identify high-risk patients, and guiding the initiation of medical therapy. By incorporating these biomarkers into clinical practice, physicians could better determine the status of sepsis and select tailored therapies for different patients, thereby achieving more precise medicine and reducing sepsis mortality. While the CAR has been proven as an effective indicator for the prognosis of sepsis, robust evidence is still insufficient to establish a specific value for the diagnosis and prognosis of sepsis. Large-scale studies with cohort designs are necessary in the future to establish a cut-off for CAR. In clinical practice, greater emphasis should be placed on patients with a high CAR.

## 4. Conclusion

Sepsis, a life-threatening disease, has several biomarkers for evaluating mortality and guiding therapy. However, due to the high costs and requirements for testing, it is imperative to identify an index that combines accuracy with convenience. The CAR has shown utility in predicting the occurrence of acute respiratory distress syndrome in patients with septic shock. It can be used as an independent predictor of all-cause mortality in severely ill patients and for prognosticating outcomes in patients with sepsis. Current findings suggest that clinicians may utilise the CAR as a bedside prognostic tool to predict mortality, assess severity, and determine the most appropriate therapy for patients with sepsis. Given its predictive value for sepsis prognosis and mortality, more focus should be directed towards the CAR in both clinical practice and future research.

## Additional Points

*Research Highlights*. (1) The C-reactive protein (CRP)/albumin ratio (CAR) is considered a more effective indicator of inflammation than CRP or albumin alone. (2) Obtaining and calculating the CAR is relatively easy. (3) The CAR demonstrates superior predictive sensitivity than CRP and is associated with a 90-day prognosis in patients with sepsis. (4) The CAR is an independent predictor of 180-day all-cause mortality in critically ill patients. (5) The CAR can aid clinicians in predicting the mortality risk in patients with sepsis.
